# Secondary exophytic glioblastoma of the cerebellopontine angle: a case report and review of the literature

**DOI:** 10.3389/fonc.2025.1660705

**Published:** 2026-01-07

**Authors:** Lirui Dai, Wenyi Zhan, Shuanghong He, Shu Jiang, Peizhi Zhou

**Affiliations:** 1 Department of Neurosurgery, West China Hospital of Sichuan University, Sichuan University, Chengdu, Sichuan, China; 2 Health Management Center, West China Hospital of Sichuan University, Sichuan University, Chengdu, Sichuan, China

**Keywords:** glioblastoma, encephalitis, surgery, cerebellopontine angle, cerebellar hemisphere, brainstem

## Abstract

**Purpose:**

Glioblastoma (GB), a World Health Organization (WHO) grade IV astrocytoma, is the most aggressive primary brain tumor in adults, with a median survival of 12–15 months despite multimodal therapy involving maximal safe resection, radiotherapy, and temozolomide. While GB predominantly arises in the supratentorial region, brainstem glioblastoma (BS-GB) represents an exceptionally rare and clinically devastating subset, accounting for only 1-2% of all GBs.

**Methods and results:**

We report a case of a 54-year-old male patient who presented with dizziness and diplopia for half a year. Examination revealed abnormal signals in the right cerebellopontine angle, cerebellar hemisphere, and brainstem. After hormone shock and antiviral treatment, the patient’s symptoms did not improve and continued to worsen. We performed a lesion biopsy on the patient. During the operation, we found a space-occupying lesion in the pontocerebellar region. The pathological examination suggested GB. After the surgery, the patient’s symptoms improved and he received regular radiotherapy and chemotherapy.

**Conclusion:**

Cases in which abnormal signals are simultaneously present in the right cerebellopontine angle, cerebellar hemisphere, and brainstem are extremely rare. After craniotomy and exploration, it was found that some of the abnormal signals were GB and some were inflammation. After surgical treatment, the patient’s symptoms were relieved. This case provides treatment ideas for patients suspected of having intracranial inflammatory lesions but who have not shown improvement despite continuous medical treatment, thereby broadening the treatment perspective.

## Introduction

Glioblastoma (GB) is the most aggressive primary brain tumor, typically arising in supratentorial regions, while its occurrence in the cerebellopontine angle (CPA) and brainstem is exceedingly rare, accounting for <1% of cases (1). The diagnostic complexity escalates when GB presents with non-specific imaging abnormalities overlapping with inflammatory or demyelinating lesions, often leading to delayed intervention (2). This case highlights a critical clinical dilemma: a patient with CPA, cerebellar, and brainstem signal abnormalities showing no response to steroid therapy, ultimately requiring surgical resection that revealed a dual pathology—GB coexisting with inflammatory changes—a phenomenon scarcely documented in literature.

The CPA region is an atypical site for GB, more frequently associated with schwannomas or meningiomas (3). When GB involves the CPA and brainstem, its infiltrative growth pattern on MRI can mimic autoimmune or infectious encephalitis, especially if steroid trials are empirically initiated (4). Notably, steroid-refractory lesions in these locations warrant early histopathological verification, as pseudo-progression or tumor-associated inflammation may confound imaging interpretation (5). Recent studies suggest that tumor microenvironment interactions could contribute to such mixed radiological-pathological presentation.

This case underscores three key challenges: 1. The diagnostic pitfalls of CPA/brainstem GB masquerading as inflammatory disease; 2. The limitations of steroid trials in atypical lesions; 3. The imperative for timely histopathological confirmation in steroid-unresponsive cases.

By integrating clinical, radiological, and pathological findings, this report aims to enhance awareness of GB’s atypical manifestations and advocate for a low threshold for biopsy in equivocal brainstem/CPA lesions.

## Case illustration

**Figure 1:**
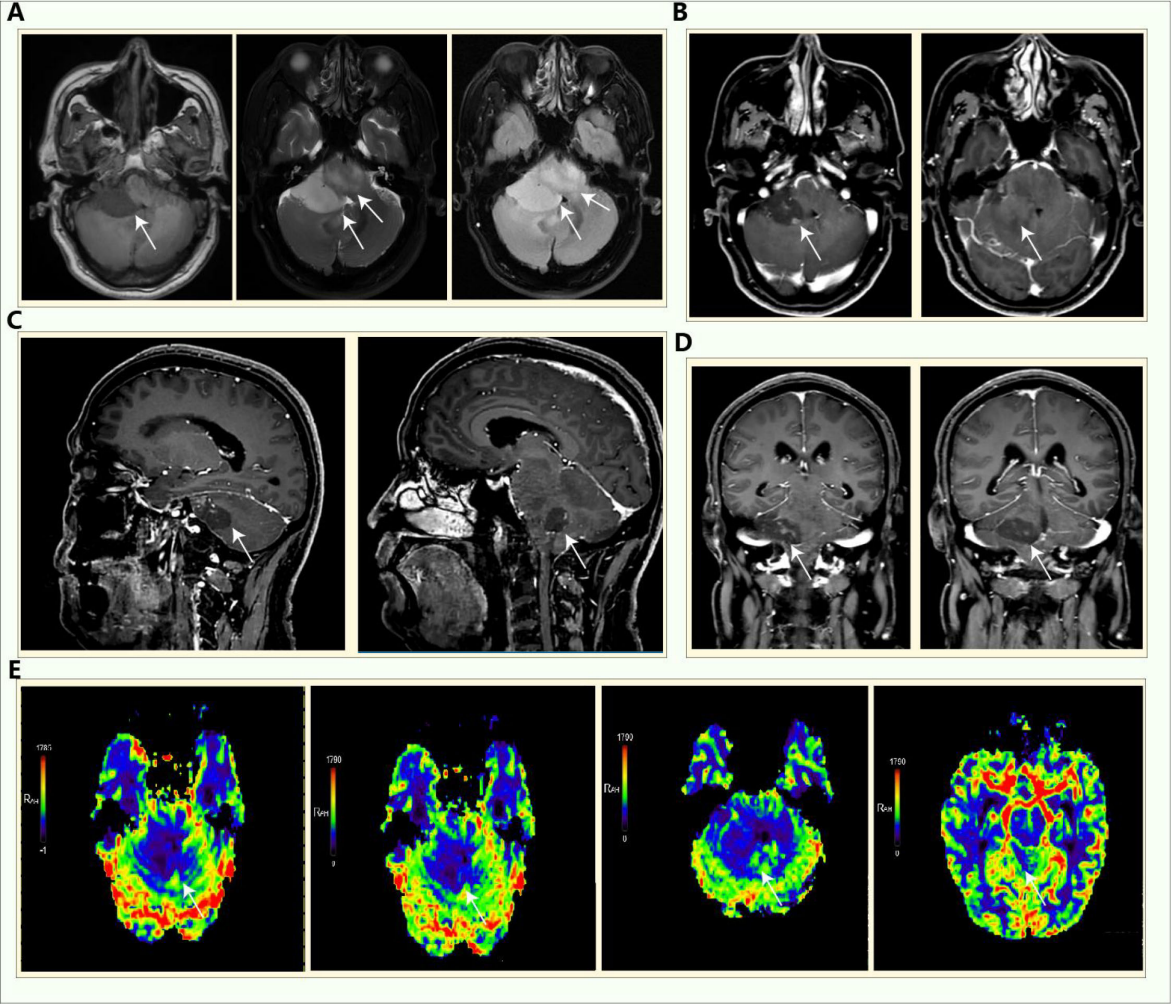
Multimodal enhanced MRI for brain tumor of the patient before the operation. **(A)** The T1, T2 and FLAIR sequences respectively; **(B-D)** the axial position, sagittal position and coronal position of the enhanced scan; **(E)** the perfusion imaging of the patient.

A 54-year-old male presented with dizziness and diplopia for the past 6 months. Lumbar puncture showed the cerebrospinal fluid was bloody and turbid, nuclear cells: 88*10^^6^, the glucose: 3.98mmol/L, the chloride: 121.5mmol/L, protein quantification: 2.69g/L. Rubella virus, cytomegalovirus and toxoplasma gondii antibody lgG were all positive. The patient regained consciousness, responded appropriately, had normal cognitive function, bilateral pupils were equal in size and round, with a diameter of approximately 3mm, had sensitive light reflexes, had double vision in both eyes, and all other neurological examinations were normal. The muscle strength and tone of the limbs were normal, the right pathological sign was positive, and the meningeal irritation sign was negative. Laboratory tests showed no obvious abnormalities or surgical contraindications. Multimodal enhanced MRI for brain tumor (**Figure 1**) indicated that in the right cerebellopontine angle area, there are patchy long T1 and slightly long T2 signals. The FLAIR sequence shows high signals, and a few vascular flow void signals can be seen within it. The boundary is still clear, with a range of approximately 36*42mm. There is no diffusion restriction. The enhanced scan shows linear and ring-shaped enhancement of the lesion, and the adjacent meninges are slightly thickened and enhanced. The cerebellum and brainstem around the lesion are not compressed. The bilateral thalamus, cerebral peduncles, brainstem, and the right cerebellar hemisphere are swollen. There are patchy slightly long T1 and T2 signals, with slightly increased FLAIR signals. No abnormal enhancement is observed. The fourth ventricle is narrowed, and the supratentorial ventricular system is slightly expanded. The brain tissue in the occipital foramen area increases. Meningoencephalitis or tumor-like lesions were considered. Antiviral, intracranial pressure reduction, hormone shock and anti-dizziness treatments were given, but the symptoms didn’t relieve and progressively worsened. After joint discussion by the neurology and neurosurgery department and communication with the patient, the patient was given a lesion biopsy. The patient had no other diseases in the past.

**Figure 2:**
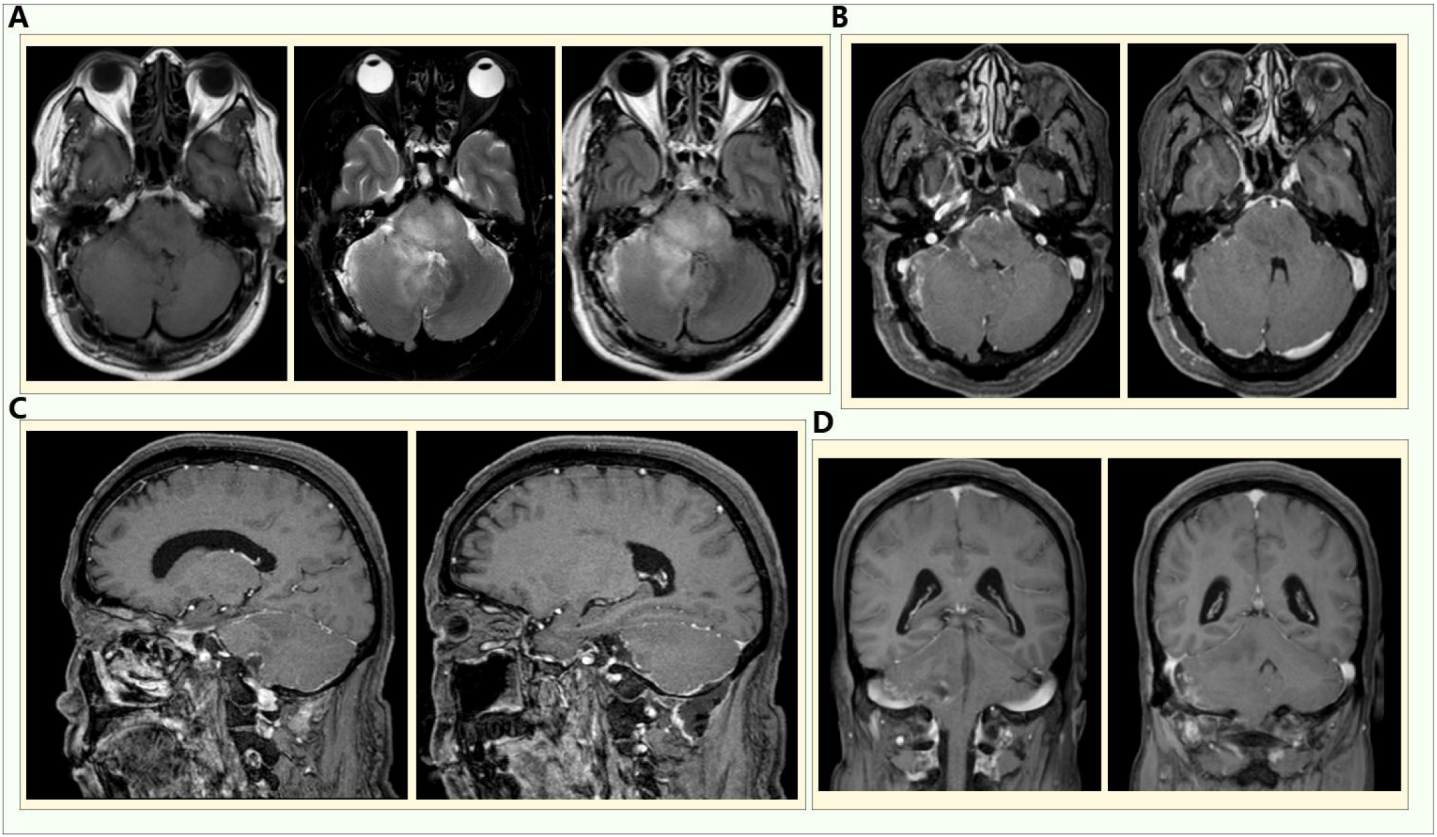
Head enhanced MRI of the patient after the operation. **(A)** The T1, T2 and FLAIR sequences respectively; **(B-D)** the axial position, sagittal position and coronal position of the enhanced scan.

We chose the right suboccipital retrosigmoid approach to perform resection of the cerebellar and brainstem occupying lesions in the right cerebellopontine angle. During the operation, an approximately 3*3*2.5cm, soft, grayish-white, gelatinous hemispherical space-occupying lesion in the right cerebellopontine angle area of the patient was found. The pedicle was located in the right pontomedullary sulcus and medipeduncle, partially affecting the cerebellar hemisphere and pons. The boundary between the lesion and the brain tissue was not clear. The upper part of the lesion was adhered to the trigeminal nerve and the rocky vein, and compressed medullary cord. The medial side of the lesion reached the abducens nerve. The blood supply to the lesion is provided by the branches of the anterior inferior cerebellar artery and the posterior inferior cerebellar artery. We removed the lesion along the boundary of the lesion. **Figure 2** showed the head enhanced MRI of the patient after the operation. The specimen was sent for histopathological examination. The immunohistochemical results indicated that the tumor cells were positive for GFAP and Oligo-2, ATRX was absent, P53 was scatteredly positive (+), the Ki67 labeling index was relatively high (about 30%), no mutations in the IDH1, IDH2, H3F3A and HIST1H3B gene-related loci were detected, no mutations in the TERT gene promoter-related loci were found, H3 K27 was diffusely positive and was in the wild-type state. Combined with the histological morphology, immunophenotype and molecular test results, it supports that this patient has glioblastoma (WHO grade 4), IDH wild-type (**Supplementary Figure S1**). The patient had no symptoms such as dizziness and blurred vision after the operation, and will receive regular radiotherapy and chemotherapy after the operation (6).

## Discussion

This case presents a rare coexistence of GB and inflammatory changes in the cerebellopontine angle (CPA), cerebellum, and brainstem, challenging both diagnosis and management. The initial steroid-refractory clinical course and ambiguous imaging findings underscore the limitations of empiric anti-inflammatory therapy in atypical posterior fossa lesions, necessitating early histopathological confirmation.

GB primarily occurs in supratentorial regions, while CPA involvement is exceptionally rare (<1% of cases) and often misdiagnosed as schwannomas or meningiomas due to overlapping radiological features (7). In our case, the infiltrative growth pattern extending into the brainstem and cerebellum further mimicked inflammatory or demyelinating diseases, such as autoimmune encephalitis or neuro-sarcoidosis (8). The lack of response to high-dose steroids, however, raised suspicion for a neoplastic process, as steroid resistance is uncommon in pure inflammatory CNS disorders. This aligns with prior reports where delayed surgical intervention due to diagnostic uncertainty resulted in worsened outcomes for brainstem GBs (9, 10).

Histopathological identification of both GB and adjacent inflammatory infiltrates highlights the complex interplay between tumor microenvironment and host immune response. GB is known to induce local astrocytosis and recruit tumor-associated macrophages (TAMs), which may contribute to steroid-unresponsive peritumoral signal changes on MRI (11–13). Notably, recent studies suggest that H3K27M-mutant gliomas (common in brainstem tumors) can trigger pronounced inflammatory reactions, potentially explaining the mixed pathology observed here (14). While our case lacked H3K27M testing (common in pediatric diffuse midline gliomas), adult brainstem GBs with similar features have been linked to NF-κB pathway activation and cytokine release (15).

The failure of steroid therapy in this case emphasizes the need for prompt biopsy or resection in steroid-refractory posterior fossa lesions. Maximal safe resection remains the cornerstone of GB management, even in high-risk areas like the CPA, as it provides diagnostic clarity and may alleviate mass effect (16). However, the proximity to cranial nerves and brainstem nuclei often limits resection completeness, necessitating adjuvant radiotherapy/temozolomide (17). Interestingly, the inflammatory component observed here raises questions about potential immune checkpoint inhibitor responsiveness, though current evidence for immunotherapy in GB is limited to supratentorial tumors (18).

Key Takeaways for Clinical Practice: 1. Steroid resistance in CPA/brainstem lesions should raise suspicion for GB, even with atypical imaging. 2. Early histopathological verification is critical to avoid delayed treatment initiation. 3. GB-associated inflammation may mimic primary inflammatory disorders, requiring integrated imaging and pathology correlation.

Further research is needed to elucidate the mechanisms underlying mixed neoplastic-inflammatory phenotypes in GB and explore targeted therapies for these aggressive variants.

## Conclusion

This case highlights the diagnostic and therapeutic challenges of glioblastoma presenting with coexisting inflammatory changes. The steroid-refractory nature of the lesion, initially mimicking an inflammatory or demyelinating process, underscores the importance of early histopathological confirmation in atypical brainstem and CPA lesions. Key lessons from this case include: 1.GB should be considered in steroid-unresponsive posterior fossa lesions, even when imaging suggests inflammatory etiologies. 2.Mixed neoplastic-inflammatory pathology may contribute to diagnostic uncertainty, necessitating a multidisciplinary approach integrating radiology, neurology, and neurosurgery. 3.Maximal safe resection remains critical for both diagnosis and symptom relief, though adjuvant therapy offers limited benefit in aggressive brainstem GBs. Given the poor prognosis of brainstem/CPA GBs, further research is needed to explore targeted therapies and immunomodulatory approaches for tumors with inflammatory microenvironments. Early molecular profiling, such as MGMT, IDH, H3K27 status, could help guide personalized treatment strategies in such rare cases.

## Data Availability

The original contributions presented in the study are included in the article/supplementary material. Further inquiries can be directed to the corresponding author.

## Supplementary material

Supplementary Figure S1 Immunohistochemistry for the indicated proteins of the patient. A: GFAP; B: P53; C:Ki67; D:ATRX; E:Oligo-2; F:H3K27M.
