# Supplementary figures and images for: Secondary exophytic glioblastoma of the cerebellopontine angle: a case report and review of the literature
Source: Front Oncol. 2026 Jan 7;15:1660705. doi: 10.3389/fonc.2025.1660705 (PMC12819263; doi:10.3389/fonc.2025.1660705)

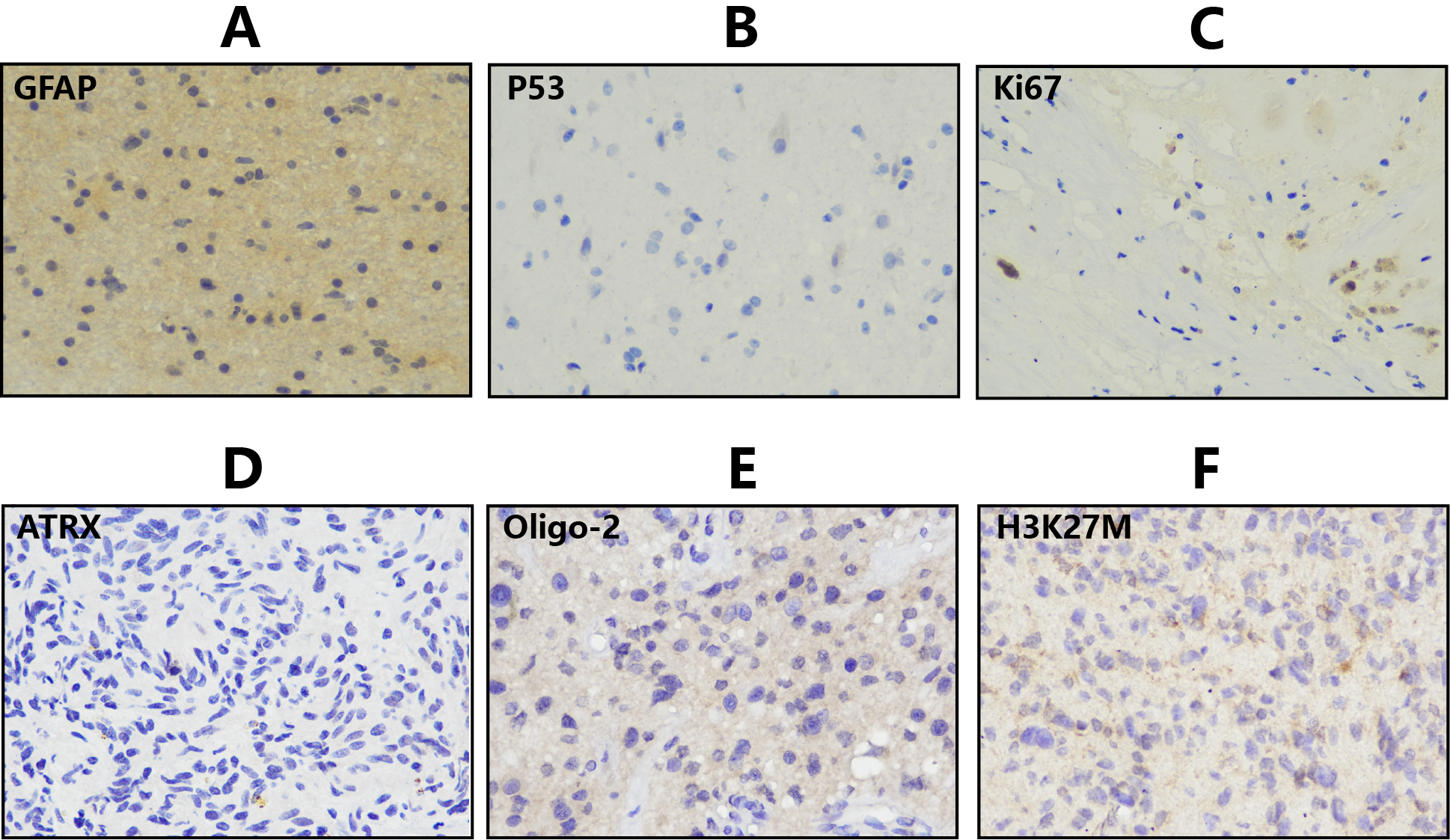

Supplement: Supplementary Figure S1 — Immunohistochemistry for the indicated proteins of the patient. A: GFAP; B: P53; C:Ki67; D:ATRX; E:Oligo-2; F:H3K27M. [file Image1.tif]
